# Efficacy and safety of the combination of anlotinib and envafolimab in the treatment of unresectable or metastatic liposarcoma: findings from a single-center retrospective study

**DOI:** 10.3389/fonc.2024.1502945

**Published:** 2025-01-10

**Authors:** Hongliang Liu, Qisheng Hao, Xi Wang, Mengxing Cheng, Fabo Qiu, Bin Zhou

**Affiliations:** ^1^ Department of Hepatobiliary and Pancreatic Surgery & Retroperitoneal Tumor Surgery, The Affiliated Hospital of Qingdao University, Qingdao, China; ^2^ Department of Oncology, Women and Children’s Hospital Affiliated to Qingdao University, Qingdao, China

**Keywords:** liposarcoma, targeted therapy, immunotherapy, efficacy, safety

## Abstract

**Objective:**

To evaluate the efficacy and safety of anlotinib combined with envafolimab in the treatment of unresectable or metastatic liposarcoma.

**Methods:**

This single-center, retrospective study enrolled 15 patients with unresectable or metastatic liposarcoma, who were treated at the Retroperitoneal Tumor Surgery Research Center of Qingdao University Affiliated Hospital between April 2022 and November 2023. The treatment regimen consisted of anlotinib combined with envafolimab. Treatment efficacy was evaluated using the Response Evaluation Criteria in Solid Tumors version 1.1. Treatment-related adverse events (TRAEs) were assessed using Common Terminology Criteria for Adverse Events version 5.0.

**Results:**

A total of 15 patients with unresectable or metastatic liposarcoma were included; among them, seven were male (46.7%) and eight were female (53.3%), with a median age of 55 years. The pathological subtype distribution was as follows: three (20.0%) patients with well-differentiated liposarcoma, 11 (73.3%) patients with dedifferentiated liposarcoma, and one (6.7%) patient with myxoid liposarcoma. At 12 weeks post-diagnosis, none of the patients achieved a complete response. The objective response rate was 6.7%, with one patient (6.7%) achieving a partial response. Disease stability was observed in 10 (66.6%) patients, which corresponded to a disease control rate of 73.3%. Disease progression occurred in four (26.7%) patients. The median follow-up time was 16.9 months and the median progression-free survival time was 14.2 months. Seven patients experienced TRAEs, of whom three (42.2%) had grade 3–4 TRAEs. The most common TRAEs were liver function abnormalities, hypertension, and fatigue.

**Conclusion:**

Anlotinib combined with envafolimab demonstrates promising efficacy and manageable safety in treating unresectable or metastatic liposarcoma.

## 1 Introduction

Liposarcoma is a rare and complex soft tissue malignancy. While surgery remains the primary treatment modality, local recurrence rates exceed 50% following surgical resection, resulting in poor patient prognosis (1). Anthracycline-based systemic chemotherapy remains the standard treatment for unresectable or metastatic disease. However, existing data suggest that targeted therapy or immunotherapy represent promising treatment alternatives. This study retrospectively investigated the efficacy and safety of anlotinib combined with envafolimab in the treatment of advanced liposarcoma.

## 2 Materials and methods

### 2.1 Clinical data and treatment protocol

**Figure 1:**
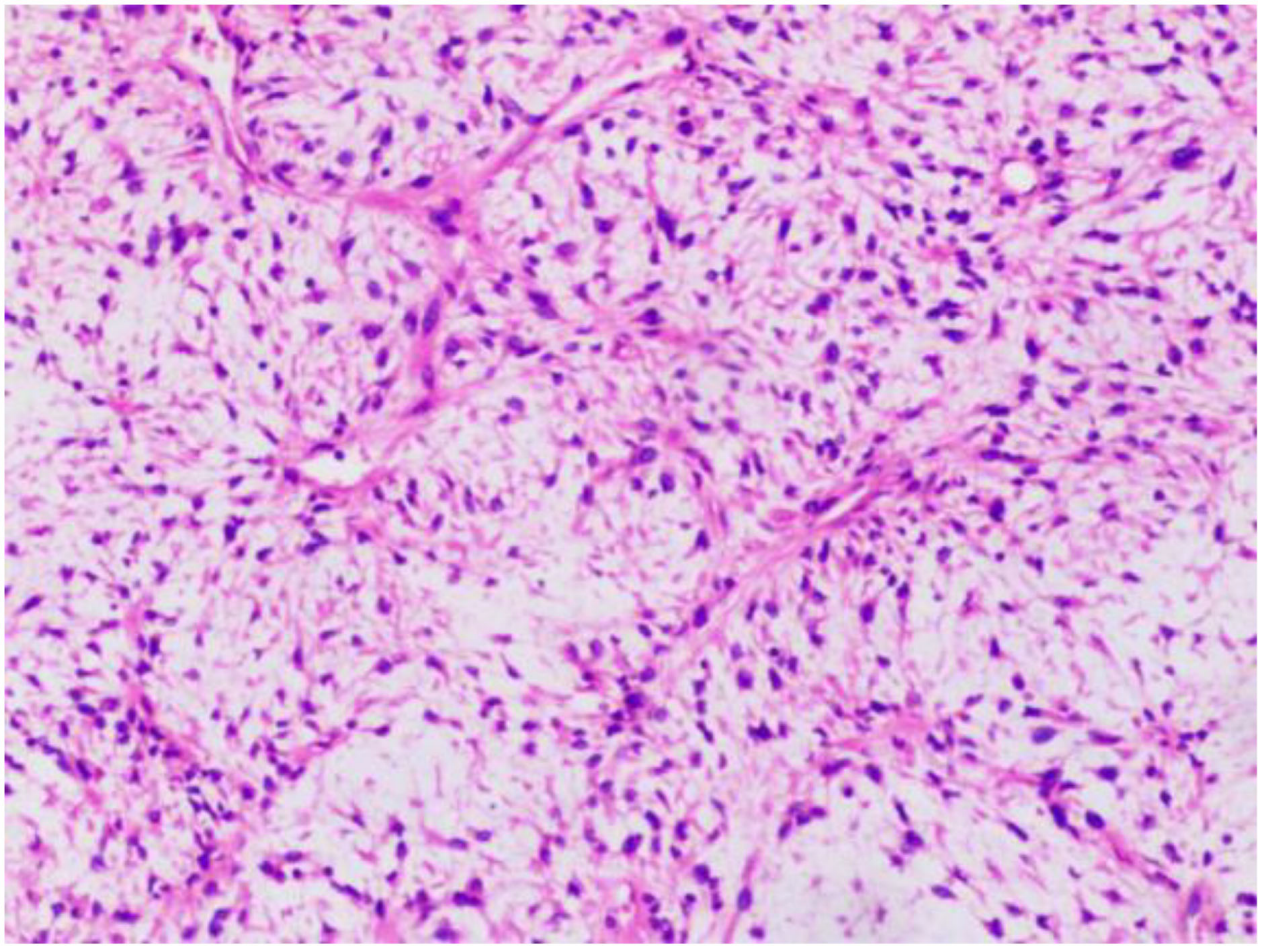
HE staining of liposarcoma.

This single-center, retrospective study included 15 patients with unresectable or metastatic liposarcoma, who were treated at the Retroperitoneal Tumor Surgery Research Center of Qingdao University Affiliated Hospital between April 2022 and November 2023. Patient information, including sex, age, treatment history, and pathological liposarcoma subtype, was collected. All the patients were pathologically diagnosed at our hospital and had complete clinical and follow-up data. The pathological diagnoses were confirmed by two senior pathologists. Representative pathology images are shown in **Figure 1**. Discussions with the multidisciplinary team led to the establishment of the following treatment protocol: 1) administer oral anlotinib (10 mg) on days 1–14; 2) administer intravenous envafolimab (200 mg) on day 1; 3) repeat the treatment cycle every 3 weeks. This study was approved by our hospital’s ethics committee (approval number: QYFY-WZLL-29433). All the patients provided informed consent.

### 2.2 Treatment efficacy evaluation

Both short-term and long-term treatment efficacy was evaluated. Short-term efficacy, including the complete response (CR), partial response (PR), stable disease (SD), progressive disease (PD), objective response rate (ORR), and disease control rate (DCR), was evaluated at 12 weeks after treatment initiation. Long-term efficacy, including the above indicators plus progression-free survival (PFS) and overall survival (OS), was assessed at the end of the follow-up period. ORR was calculated as: (CR + PR)/total number of cases × 100%. DCR was calculated as: (CR + PR + SD)/total number of cases × 100%. PFS was defined as the time from treatment initiation to disease progression or the last follow-up date if progression had not occurred. OS was defined as the time from treatment initiation to death from any cause or the last follow-up date for surviving patients. All patients underwent imaging examinations at baseline (prior to treatment initiation) and after every two treatment cycles. Treatment efficacy was assessed according to the RECIST 1.1 criteria.

### 2.3 Safety assessment

Treatment tolerance was evaluated by monitoring adverse events. Patients who tolerated the treatment continued to adhere to the original protocol; those that experienced adverse events, discontinued the treatment. All adverse reactions were graded according to the Common Terminology Criteria for Adverse Events (CTCAE version 5.0).

### 2.4 Follow-up

Regular telephone follow-up interviews were conducted to collect PFS and OS data.

### 2.5 Statistical analysis

Data analysis was performed using SPSS 24.0 statistical software. All 15 eligible patients were included in the analyses. Continuous variables were reported as median (range). Categorical variables were presented as frequency counts and percentages. Survival analysis was performed using the Kaplan-Meier method, with a significance level of α <0.05.

## 3 Results

### 3.1 Patient characteristics

A total of 15 patients were enrolled in this study; among them, three patients had WDLPS, 11 patients had DDLPS, and one patient had MLPS. Seven of the patients were male and eight were female. The median age at diagnosis was 55 years (range: 41–75 years). All patients had primary tumors that were located in the retroperitoneal space. Four patients had unresectable disease at initial diagnosis, six had local recurrence, and five had distant metastases. Of the 15 patients, 11 had a history of previous liposarcoma resection.

In terms of previous treatment history, four patients received anlotinib monotherapy, three received anlotinib combined with chemotherapy, and four received anlotinib combined with radiotherapy. The remaining four patients had no prior treatment history. All three patients who previously received chemotherapy had DDLPS and were treated with ifosfamide (7.5 mg/m²/cycle) combined with doxorubicin (75mg/m²/cycle). Of the four patients who received radiotherapy, three had DDLPS and one had MLPS. Radiation was administered to the retroperitoneal region, with external beam radiation doses of 95% PTV 45–50 Gy (1.8–2.0 Gy/fraction). The patient baseline characteristics are summarized in **Table 1**.

**Table 1:** Basic information and clinical features of enrolled liposarcoma patients.

Clinical features	Proportion (%)
Age	
Range of variation	41-75
Median age	55
Sex	
Male	7 (46.7%)
Female	8 (53.3%)
Pathological classification	
WDLPS	3 (20%)
DDLPS	11 (73.3%)
MLPS	1 (6.7%)
Previous liposarcoma surgery history	
Yes	11 (73.3%)
No	4 (26.7%)
Invading surrounding organs	
Yes	10 (66.7%)
No	5 (33.3%)
History of previous drug treatment	
Without antitumor therapy	4 (26.7%)
Anlotinib monotherapy	4 (26.7%)
Anlotinib combined with chemotherapy	3 (20%)
Anlotinib combined with radiotherapy	4 (26.7%)
Single or multiple lesion	
Single lesion	6 (40%)
Multiple lesion	9 (60%)
Pathological characteristics	
MDM2 (+)	14 (93.3%)
CDK4 (+)	14 (93.3%)
FUS-DDIT3 (+)	1 (6.7%)

### 3.2 Treatment efficacy and patient prognosis

**Figure 2:**
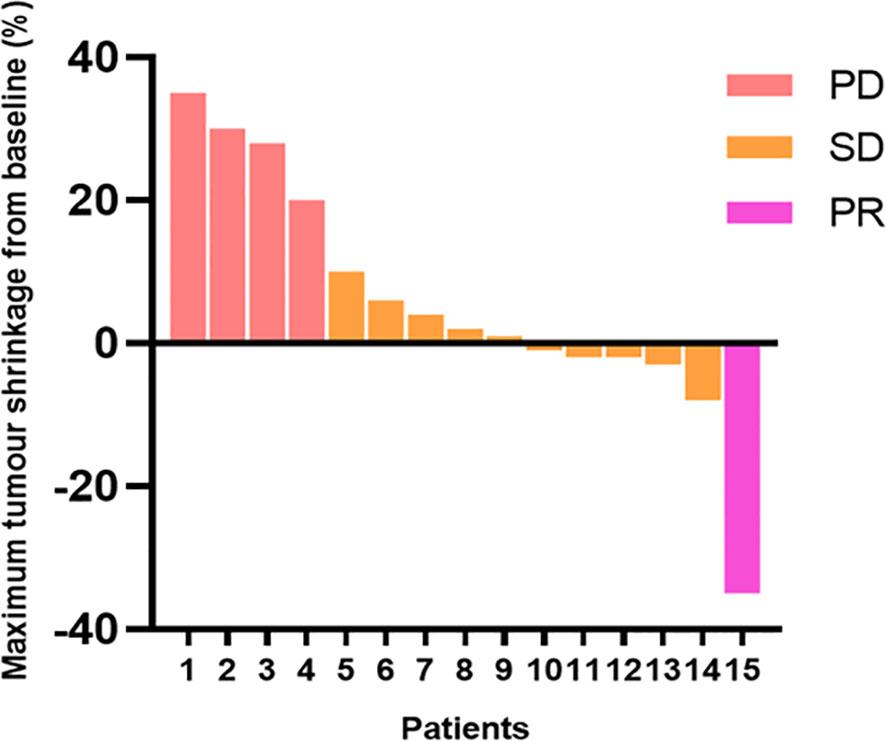
The extent to which each enrolled patient responded to this regimen.

**Figure 3:**
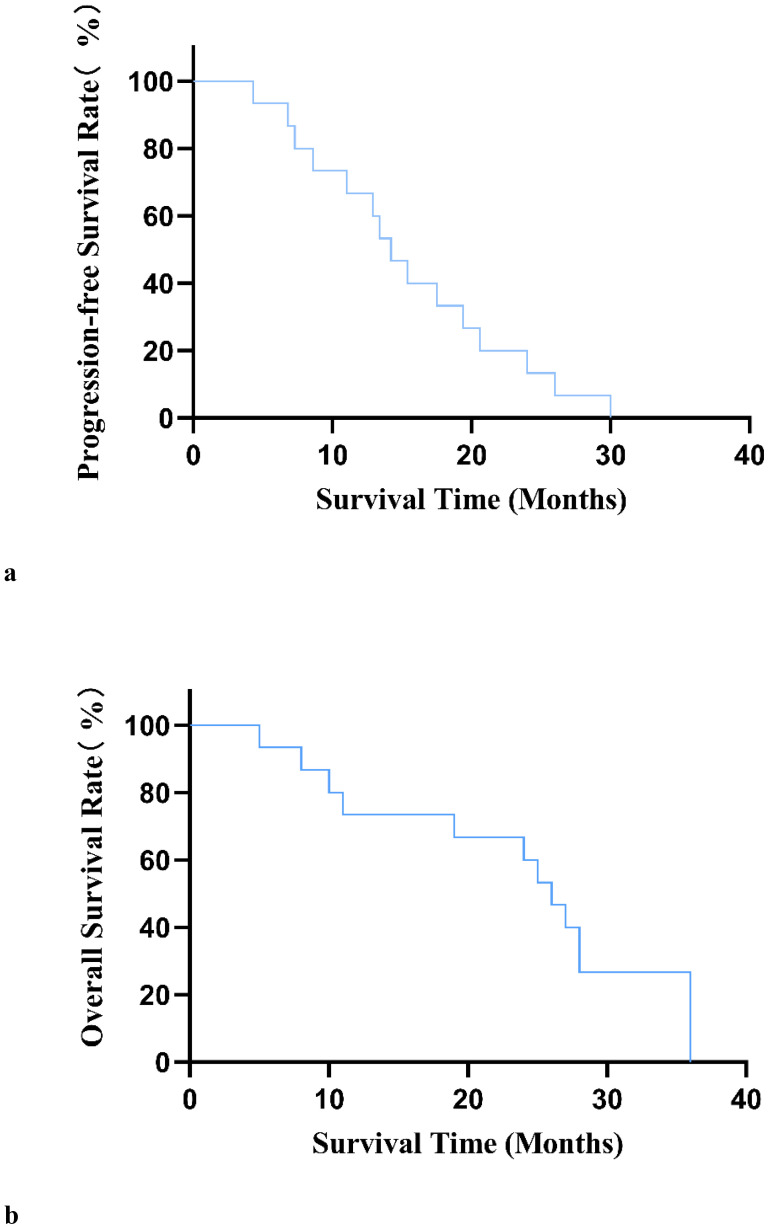
**(A)** Progression-free survival curve in 15 patients with unresectable or metastatic liposarcoma. **(B)** Overall survival curve of 15 patients with unresectable or metastatic liposarcoma.

After 12 weeks of treatment with anlotinib plus envafolimab, none of the patients achieved a CR; however, one patient (6.7%) achieved a PR, 10 patients (66.6%) had SD, and four patients (26.7%) had PD. The DCR reached 73.3%, and the ORR was 6.7%. Among the 11 patients in the DDLPS group, one (9.1%) achieved a PR, seven (63.6%) had SD, and three (27.3%) had PD. Of the three patients in the WDLPS group, two had SD and one had PD. The individual patient responses are shown in **Figure 2**. As of April 2024, four patients had died. The median follow-up time was 16.9 months (range: 4.1–22.3 months), with a median PFS (mPFS) of 14.2 months (95% confidence interval [CI]: 11.1–17.4 months). The median OS (mOS) was 26 months (95% CI: 22.2–29.7 months) (**Figures 3A, B**).

### 3.3 Adverse events

During treatment, seven patients experienced treatment-related adverse events (TRAEs); three of whom (42.2%) had grade 3–4 TRAEs (**Table 2**). The most common TRAEs were liver function abnormalities, hypertension, and fatigue. As all of the adverse reactions were manageable, no dose adjustments or treatment interruptions were required.

**Table 2:** General situation of adverse reactions.

Adverse reaction	Level I	Level II	Level III	Level IV	Total
Abnormal liver function	2	1	1	1	5
Hypertension	2	2	1	0	5
Fatigue	1	0	1	2	4
Gastrointestinal reaction	1	0	2	0	3
Pneumonia	1	1	0	0	2
Oral mucositis	1	1	0	0	2
Albuminuria	1	1	0	0	2
Anemia	0	0	1	0	1
Thrombocytopenia	0	0	1	0	1
Hypothyroidism	0	0	0	0	0
Hyperthyroidism	0	0	0	0	0

## 4 Discussion

Liposarcoma accounts for ~15%–20% of soft tissue sarcomas (STSs) (2). It is a rare malignant tumor that arises due to dysregulated lipocyte differentiation, and develops primarily in the extremities and retroperitoneum (representing 41% and 36% of cases, respectively) (3). According to the WHO Classification of Soft Tissue Tumors (5^th^ edition, 2020) (4), liposarcoma subtypes include: atypical lipomatous tumor (ALT)/well-differentiated liposarcoma (WDLPS), dedifferentiated liposarcoma (DDLPS), myxoid liposarcoma (MLPS), pleomorphic liposarcoma (PLPS), and myxoid pleomorphic liposarcoma (MPLPS), which has been newly added to this latest edition.

While surgical resection remains the primary treatment for all liposarcoma subtypes, therapeutic options are limited and outcomes are inconsistent for patients with advanced/unresectable disease. Current first-line systemic therapy consists of doxorubicin, ifosfamide, or their combination (5). However, treatment response varies significantly among liposarcoma subtypes. Moreover, the median survival time of patients with advanced, albeit, chemotherapy-sensitive subtype, is only 2 years (6). Novel agents such as trabectedin have shown promise in 3D culture models (7); however, they are still in the exploratory phase of development. Therefore, there is an urgent need for new drugs or therapeutic strategies with the potential to improve the current treatment landscape.

Existing data suggest that targeted therapy or immunotherapy may increase treatment responses. Studies involving DDLPS patients have shown that the sequential administration of the CDK4 inhibitor palbociclib combined with lenvatinib can achieve synergistic effects (8). Additionally, research indicates that liposarcoma has a denser microvascular network than other sarcoma subtypes, suggesting that it may be especially sensitive to anti-angiogenic therapy (9). In a study by Li et al., anlotinib was used to treat 40 patients with STS who were not eligible for chemotherapy. The median PFS was 6.83 months, and the median OS was 27.40 months. One patient achieved a PR and 26 patients had SD, which resulted in a DCR of 67.5% (27/40) (10). In the ALTER-0202 study, 13 patients with recurrent/metastatic advanced liposarcoma receiving anlotinib had a 12-week PFS rate (PFR) of 63%, with an mPFS and an mOS of 5.6 and 13 months, respectively (11). Meanwhile, in the ALTER-S006 study, 49 STS patients who achieved a PR or SD after receiving at least four cycles of first-line anthracycline-based chemotherapy underwent maintenance therapy with anlotinib. The over cohort had an mPFS of 9.1 months, with liposarcoma patients having an mPFS of 12.5 months (12). In another retrospective study of 17 patients with metastatic/recurrent liposarcoma who were treated with anlotinib, the mPFS was 27.9 weeks, with a 24-week PFR of 58.8% and an OS of 56.6 weeks (13). These studies consistently demonstrate the favorable efficacy of anlotinib as an anti-angiogenic treatment for liposarcoma. These promising results have led to the inclusion of anlotinib in the CSCO guidelines as a second-line treatment option for STSs (14).

Beyond anti-angiogenic agents, immunotherapy has shown proven efficacy against various solid tumors, including STSs. STSs are traditionally considered as “immunologically inert or cold” tumors, characterized by low-level immune infiltration and poor immune reserves. As such, STSs generally respond poorly to immunotherapy. However, recent biomarker studies have revealed significant immune heterogeneity among different sarcoma subtypes, meaning that immunotherapy tailored to specific biomarker profiles and tissue subtypes shows promise in improving the treatment outcomes of patients with STSs (15). From an immunogenomics perspective, sarcomas with complex karyotypes are more likely to be “immunologically hot”. This genomic complexity translates to increased tumor mutational burden (TMB) and a tumor microenvironment (TME) that facilitates immune cell infiltration. These characteristics may increase the responsiveness of sarcomas to immunotherapy. Multiple clinical trials have investigated various immunotherapeutic approaches, including immune checkpoint inhibitors (ICIs), therapeutic antibodies, cancer vaccines, immunomodulators, adoptive cell therapy, and T-cell-receptor-engineered T cell therapy, for the treatment of STSs. Among these, ICIs are the most widely used. However, clinical trials of ICI monotherapy have yet to demonstrate convincing clinical benefits for patients with STSs. The initial results of a multicenter phase II study (SARC028) of pembrolizumab (200 mg every 3 weeks) in patients with advanced STS were encouraging, with 2/10 liposarcoma patients achieving a PR (16). However, in the expanded liposarcoma cohort, the ORR was only 10%, with an mPFS of 2 months and a 12-week PFR of 44%; these low response rates led to the study failing to meet its predetermined endpoint (17). A 2020 meta-analysis of clinical trials investigating the utility of PD-1 or PD-L1 antagonists in the treatment metastatic STS. In the nine trials included, the 153 patients (39.8%) who received PD1/PD-L1 antagonist monotherapy had an ORR of 15.1% (18). Furthermore, among the 61 patients with retroperitoneal DDLPS, the ORR was only 7.3% (19). In the Alliance A091401 study, no DDLPS patients responded to treatment in either the nivolumab monotherapy group or the nivolumab plus ipilimumab combination group (20).

Given the limited efficacy of anti-angiogenic drugs as a monotherapy for liposarcoma, and the similarly disappointing results generated with immunotherapy alone, optimizing treatment strategies remains a key focus for researchers in this field. Combining immunotherapy with chemotherapy, radiotherapy, or targeted therapy can potentially transform “cold” liposarcoma tumors into “hot” ones (21). Multiple studies have demonstrated the synergistic effects of combining anti-angiogenic drugs with chemotherapy and immunotherapy. For instance, anti-angiogenic targeted drugs that block the VEGF signaling pathway can be used to address the challenge of suboptimal anti-tumor immune responses in patients with sarcomas. Such a strategy aims to reduce hypoxia, while promoting drug delivery and immune cell infiltration into the TME, which ultimately modulates host immunity and sensitizes it to immunotherapy (3, 22). Furthermore, using a combination therapy can overcome tumor resistance (which is a common limitation of monotherapy) and achieve higher response rates through synergistic effects. The success of combination therapies has been demonstrated in the context of lung cancer, hepatocellular carcinoma, and renal cancer (23).

Several clinical studies have investigated combination therapies against liposarcoma. For instance, one study treated 47 patients with retroperitoneal liposarcoma using a combination of eribulin, amlotinib and camrelizumab over a median follow-up period of 21.8 months (24). The ORR and DCR were 18.2% and 75%, respectively. In another study by the same team, 57 patients with RST received a combination of anlotinib and camrelizumab. Two (3.5%) patients achieved a CR and 13 (22.8%) patients a PR, with an ORR and a DCR of 26.3% and 80.7%, respectively (25). A retrospective study of 24 patients with advanced DDLPS receiving chemotherapy combined with a PD-1 inhibitor and anlotinib, reported an ORR of 20.8% and a DCR of 83.3% over a median follow-up time of 7.7 months (26).The multicenter, single-arm phase I/II ALTER-S007 clinical trial evaluated the efficacy of penpulimab combined with anlotinib and epirubicin as a first-line treatment for unresectable/metastatic STS. The recommended phase II dose was determined as: anlotinib (10 mg, days 1–14), epirubicin (60 mg/m², day 1), and penpulimab (200 mg, day 1), repeated every 3 weeks. The grade 3–4 adverse events were primarily epirubicin-related hematological toxicities, with no increased TRAE risk observed on addition of anlotinib and penpulimab. Among the seven evaluable patients in the early phase of the trial, three achieved a PR and four achieved SD, with all patients reaching the goal DCR (27). A retrospective study of camrelizumab combined with anlotinib and eribulin in 60 patients with metastatic retroperitoneal LPS/LMS (including 38 liposarcoma cases: nine with WDLPS, 24 with DDLPS, five with MLPS) reported an ORR and a DCR of 19.4% and 72.2%, respectively (28). These data demonstrate the promising potential of combining targeted therapy and immunotherapy for the treatment of liposarcoma, particularly DDLPS. However, significant variations exist among studies in terms of patient populations and treatment protocols. Moreover, the inherent heterogeneity of liposarcoma contributes to the variability of the results. Further studies are needed to determine the optimal therapeutic combinations and their value in clinical practice.

In the present study, we conducted a preliminary investigation into the efficacy and safety of anlotinib combined with envafolimab in patients with unresectable/metastatic liposarcoma at our treatment center. The results showed that only one (6.7%) patient achieved a PR, yielding an ORR of 6.7%. Meanwhile, 10 (66.6%) patients achieved SD, which corresponded to a DCR of 73.3%. There results were consistent with previous findings. Although the ORR was not satisfactory, the combination of targeted therapy and immunotherapy appeared to show advantages over the respective monotherapies at improving disease control. Moreover, in view of the fact that this study is an exploration of a new combination therapy regimen in clinical practice and the common drug intolerance in patients with advanced tumors, in order to minimize the incidence of serious adverse reactions, the dose specification of 10mg was selected.In terms of results, the safety profile of the combination therapy was similar to that reported by previous studies (29–31), with all adverse events being manageable.Patients ultimately benefitted, and we can explore larger sized drug dosages in patients in the future.

The present study had several limitations. Due to its single-center, retrospective nature, this study provides only preliminary insights into the efficacy and safety of anlotinib combined with envafolimab in treating unresectable or metastatic liposarcoma. The rationale for focusing specifically on liposarcoma rather than several STS types was to reduce the influence of confounding factors. However, due to the rarity of this disease, our sample size was relatively small, which may have introduced some bias into the results. Moreover, the evaluation was limited to clinical efficacy. Future multi-center, large-sample, prospective clinical trials are warranted to validate our findings and identify specific prognostic biomarkers.

In conclusion, as liposarcoma research continues to advance, differences in TME characteristics and pathogenic mechanisms among the STS subtypes will be revealed. Therefore, subtype-specific treatment approaches will likely become the primary focus of future research endeavors. Ultimately, as it seems unlikely that a single, universal therapy for STS will emerge, patient selection based on factors such as histological subtype, TME characteristics, immune category, and tumor-infiltrating lymphocyte profile, will be essential.

## Data Availability

The original contributions presented in the study are included in the article/supplementary material. Further inquiries can be directed to the corresponding authors.
